# Continuous chromatin state feature annotation of the human epigenome

**DOI:** 10.1093/bioinformatics/btac283

**Published:** 2022-04-22

**Authors:** Habib Daneshpajouh, Bowen Chen, Neda Shokraneh, Shohre Masoumi, Kay C Wiese, Maxwell W Libbrecht

**Affiliations:** School of Computing Science, Simon Fraser University, Burnaby, BC V5A 1S6, Canada

## Abstract

**Motivation:**

Segmentation and genome annotation (SAGA) algorithms are widely used to understand genome activity and gene regulation. These methods take as input a set of sequencing-based assays of epigenomic activity, such as ChIP-seq measurements of histone modification and transcription factor binding. They output an annotation of the genome that assigns a chromatin state label to each genomic position. Existing SAGA methods have several limitations caused by the discrete annotation framework: such annotations cannot easily represent varying strengths of genomic elements, and they cannot easily represent combinatorial elements that simultaneously exhibit multiple types of activity. To remedy these limitations, we propose an annotation strategy that instead outputs a vector of chromatin state features at each position rather than a single discrete label. Continuous modeling is common in other fields, such as in topic modeling of text documents. We propose a method, *epigenome-ssm-nonneg*, that uses a non-negative state space model to efficiently annotate the genome with chromatin state features. We also propose several measures of the quality of a chromatin state feature annotation and we compare the performance of several alternative methods according to these quality measures.

**Results:**

We show that chromatin state features from *epigenome-ssm-nonneg* are more useful for several downstream applications than both continuous and discrete alternatives, including their ability to identify expressed genes and enhancers. Therefore, we expect that these continuous chromatin state features will be valuable reference annotations to be used in visualization and downstream analysis.

**Availability and implementation:**

Source code for *epigenome-ssm* is available at https://github.com/habibdanesh/epigenome-ssm and Zenodo (DOI: 10.5281/zenodo.6507585).

**Supplementary information:**

Supplementary data are available at *Bioinformatics* online.

## 1 Introduction

Sequencing-based genomic assays can measure many types of genomic biochemical activity, including transcription factor binding, chromatin accessibility, transcription and histone modifications. Data from sequencing-based genomic assays are now available from hundreds of human cellular conditions, including varying tissues, individuals, disease states and drug perturbations.

Segmentation and genome annotation (SAGA) methods are widely used to understand genome activity and gene regulation (Libbrecht *et al.*, 2021). These algorithms take as input a collection of sequencing-based genomic datasets from a particular tissue. They output an annotation of the genome that assigns a label to each genomic position. They are unsupervised; they discover categories of activity (such as promoters, enhancers, genes, etc.) without any prior knowledge of known genomic elements and a human interprets these categories, similar to a clustering algorithm. Many SAGA methods have been proposed, including HMMSeg (Day *et al.*, 2007), ChromHMM (Ernst and Kellis, 2012; Ernst *et al.*, 2011), Segway (Hoffman *et al.*, 2012b) and others. See below for a comprehensive review of previous work.

All existing SAGA methods output a discrete annotation that assigns a single label to each position. This discrete annotation strategy has several limitations. First, discrete annotations cannot represent the strength of genomic elements. Variation among genomic elements in intensity or frequency of activity of cells in the sample is captured in variation in the intensity of the associated marks. Such variation is lost if all such elements are assigned the same label. In practice, SAGA methods often output several labels corresponding to the same type of activity with different strengths, such as ‘Promoter’ and ‘WeakPromoter’ (Ernst and Kellis, 2012; Hoffman *et al.*, 2012a). Second, a discrete annotation cannot represent combinatorial elements that simultaneously exhibit multiple types of activity. To model combinatorial activity, a discrete annotation must use a separate label to represent each pair (or triplet, etc.) of activity types. For example, intronic enhancers usually exhibit marks of both transcription and regulation (Hoffman *et al.*, 2012a). However, representing all possible combinations of activity types with discrete labels would require a number of labels that grows exponentially in the number of activity types.

**Fig. 1.**
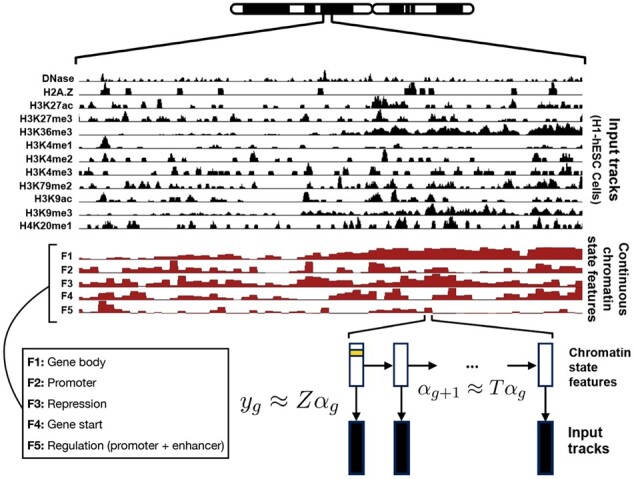
Overview of continuous chromatin state feature annotation and *epigenome-ssm*. *epigenome-ssm* takes as input a set of genomic assays, each represented as a real-valued track over the genome (12 tracks). It outputs a set of real-valued chromatin state features for each position in the genome (five tracks), using a state space model. Each chromatin state feature putatively represents a different type of activity. These continuous features can represent multiple types of activities. For example, feature 1 marks gene bodies and feature 5 marks general regulatory activity which includes both promoters and enhancers (Section 4).

In this work, we propose a continuous genome annotation strategy. That is, our method takes as input a set of sequencing-based genomic data tracks and outputs a vector of real-valued *chromatin state features* for each genomic position, where each chromatin state feature putatively represents a different type of activity (Fig. 1). Continuous chromatin state features have a number of benefits over discrete labels. First, chromatin state features preserve the underlying continuous nature of the input signal tracks, so they preserve more of the information present in the raw data. Second, in contrast to discrete labels, continuous features can easily capture the strength of a given element. Third, chromatin state features can easily handle positions with combinatorial activity by assigning a high weight to multiple features. Fourth, chromatin state features lend themselves to expressive visualizations because they project complex datasets onto a small number of dimensions that can be mapped to axes of a plot. For these reasons, in other fields, continuous modeling is often preferred over discrete. For example, the widely used method of topic modeling for text documents assigns a continuous weight to each of a number of categories (such as ‘sports’ or ‘politics’) for each document (Landauer *et al.*, 2013).

In this work, we explore the utility of chromatin state feature annotation. We propose several measures of the quality of a chromatin state feature annotation and we compare the performance of several alternative methods according to these quality measures. We propose a non-negative state space model for this problem, *epigenome-ssm-nonneg*, that produces the highest-quality continuous annotations of the methods we compared.

## 2 Related work

Many methods have been proposed for discrete chromatin state label annotation [reviewed in Libbrecht *et al.* (2021)] (Coetzee *et al.*, 2018; Day *et al.*, 2007; Ernst and Kellis, 2012; Hoffman *et al.*, 2012b; Larson *et al.*, 2013; Mammana and Chung, 2015; Sohn *et al.*, 2015; Zhou and Troyanskaya, 2016). The primary model used by these methods is the hidden Markov model (HMM). An HMM is a probabilistic model that assumes that there is a latent (unknown) chromatin state label at each position, that the observed genomic datasets are generated as a function of this label and that the label at position *i* depends on the label at position *i* *−* 1. Later work extended this basic approach in a number of ways. First, there are three methods for modeling the input genomic data: one can binarize the data and model 0/1 values with a Bernoulli distribution (Ernst and Kellis, 2012), use a continuous measure of signal strength such as fold enrichment over control modeled with a Gaussian distribution (Hoffman *et al.*, 2012b; Sohn *et al.*, 2015), or model raw read counts with a negative Binomial distribution (Mammana and Chung, 2015). Second, some methods (Hoffman *et al.*, 2012b) use statistical marginalization to handle unmappable regions. Third, several strategies exist for modeling segment lengths or for producing annotations on multiple length scales (Hoffman *et al.*, 2012b; Larson *et al.*, 2013). Finally, several strategies have been proposed to guide the choice of the number of labels (Coetzee *et al.*, 2018; Sohn *et al.*, 2015; Zhang *et al.*, 2016; Zhou and Troyanskaya, 2016).

A related class of joint annotation methods aim to improve epigenome annotations by simultaneously annotating many cell types and sharing position-specific information between the annotations (Biesinger *et al.*, 2013; Dsouza *et al.*, 2021; Libbrecht *et al.*, 2015a,b; Zhang *et al.*, 2016; Zhang and Hardison, 2017). Such joint annotations can be more accurate, but have the drawback that they may mask differences among cell types. We do not consider the joint annotation task in this work, but adapting the continuous annotation approach to this task is a promising direction for future work.

Another related task aims to take data from all available cell types as input to produce a single pan-cell-type (as opposed to cell-type-specific) annotation, a task sometimes known as ‘stacked’ annotation. Several methods have been proposed to produce a discrete (Hoffman *et al.*, 2012a; Libbrecht *et al.*, 2019) and continuous (Dsouza *et al.*, 2021; Durham *et al.*, 2018; Schreiber *et al.*, 2020) pan-cell-type annotations. However, these methods do not apply to the cell-type-specific case, so we do not compare to these methods below.

## 3 Materials and methods

### 3.1 Reference genome

We performed all analyses using the human reference genome hg19. To improve computational efficiency, following previous work (Ernst and Kellis, 2012; Libbrecht *et al.*, 2019), we divided the genome into 200 bp bins and performed all analysis at the bin level. In order to reduce the computational time, we followed Segway (Hoffman *et al.*, 2012b) and trained our model using just the ENCODE Pilot regions (ENCODE, 2020), which cover about 1% of the human genome. We applied the trained model to annotate chromatin state features across the whole genome. We also removed the ENCODE blacklist regions from all analysis.

### 3.2 Epigenomic datasets

We downloaded epigenomic datasets from the Roadmap Epigenomics data portal (Kundaje *et al.*, 2015). See Kundaje *et al.* (2015) for a full description of the data processing pipeline. Briefly, reads were mapped to the reference genome, shifted and extended according to the fragment length and compared to an input control. We represented the signal at a given position as the fold-enrichment ratio of the observed read count compared to input (Kundaje *et al.*, 2015). To reduce the influence of large outliers, following previous work (Hoffman *et al.*, 2012b), we transformed ChIP-seq values using the transformation $\text{arcinsh}(x)=\text{log}\;(x+\sqrt{\left(x^{2}+1\right)})$. We used a representative set of data from 12 assays and eight cell types (Supplementary Table S1).

### 3.3 State space model

We developed a state space model (SSM) (Durbin and Koopman, 2012) for annotating the genome with chromatin state features. This model takes as input a vector of *E* observed genomic datasets for each position, $y_{g}\in {\mathbb{R}}^{E}$, for g∈1…G where *G* is the length of the genome. This model assumes that at position *g* there is a latent vector $\alpha_{g}\in {\mathbb{R}}^{K}$ that encodes the chromatin state features of that position. It assumes that the observed data vector at that position *y_g_* is generated as a linear function of *α_g_* parameterized by the emission matrix *Z* plus Gaussian noise, $y_{g}=Z\alpha_{g}+\epsilon_{g}\;\;\epsilon_{g}∼N(0,I)$. It further assumes that the latent vector $\alpha_{g+1}$ is generated as a linear function of *α_g_* parameterized by the transition matrix *T* plus Gaussian noise, $\alpha_{g+1}=T\alpha_{g}+v_{g}\;\;v_{g}∼N(0,I)$. To learn the SSM model, we use the expectation–maximization (EM) algorithm to maximize the log likelihood of the model as a function of its parameters, $Z\in {\mathbb{R}}^{E\times K}$ and $T\in {\mathbb{R}}^{K\times K}$. Briefly, this algorithm alternates two steps, the E step and the M step. In the E step, we hold *Z* and *T* fixed and use a message-passing algorithm to efficiently estimate $\alpha_{1:g}$ and compute sufficient statistics for updates to *Z* and *T*. In the M step, we use these sufficient statistics to update *Z* and *T*. We initialized $Z∼\text{Uniform}{\left(0,1\right)}^{E\times K}$ and *T* = *I_K_*.

To limit the model’s capacity to overfit and its sensitivity to local optima, we additionally add two *L*_2_ regularization terms to the optimization’s objective function *J*(*Z*, *T*), which encourage *Z* and *T* to have small values: minimize $J(Z,T)=\text{log}\;P(\alpha ,Z,T|Y)+\lambda_{1}||Z|{|}_{F}+\lambda_{2}||T|{|}_{F}$.

### 3.4 Non-negativity constraint

We developed a version of our model, *epigenome-ssm-nonneg*, in which the chromatin state features *α_g_* and the emission parameters *Z* are both constrained to be non-negative. To optimize *Z* under this constraint, we used an active set method of Lagrange multipliers to enforce the non-negativity constraint (Gupta and Hauser, 2007). Specifically, we add a Lagrange multiplier term to our objective function minimize $J_{\Lambda }(Z,T,\Lambda_{Z},\Lambda_{\alpha })=J(Z,T)+\text{tr}(\Lambda_{Z}^{T}Z)$. The active set method takes advantage of the property of complementary slackness: $\Lambda_{Z}{}_{e,m}Z_{e,m}=0$. At each iteration, we maintain a list of parameters with non-negative Lagrange multipliers. Parameters are added or removed from the active set when the optimization assigns them a zero or non-zero value respectively. At each iteration, we update the Lagrange multipliers associated with each parameter in the active set in order to stop the parameters from becoming negative.

For *α*, we enforce the non-negativity constraint by projecting the optimized state to lie in the constraint space

(1) $$\begin{array}{c} {\hat{\alpha }}_{g|g}^{P}=\underset{\alpha }{\text{argmin}}\{{\left(\alpha -{\hat{\alpha }}_{g|g}\right)}^{T}(\alpha -{\hat{\alpha }}_{g|g}):A\alpha =0\}\\ A_{ij}=1\;\;\text{iff}\;\;i=j\;\text{and}\;{\hat{\alpha }}_{g|g}<0\;\;\text{otherwise}\;A_{ij}=0 \end{array},$$

where ${\hat{\alpha }}_{g|g}^{P}$ is the constrained value, and ${\hat{\alpha }}_{g|g}$ is the unconstrained update estimate at time *g*. Given the optimized state ${\hat{\alpha }}_{g|g}^{P}$, we construct the constraint matrix *A* as Equation (1) and make *b* as a zero vector. Then, the best constrained estimate is given by ${\hat{\alpha }}_{g|g}^{P}=\hat{\alpha_{g|g}}-A^{T}{\left(AA^{T}\right)}^{-1}(A{\hat{\alpha }}_{g|g}-b)$. To apply the non-negativity constraint on the emission matrix *Z*, we similarly used a Lagrange multiplier active set method as follows. Each update has two steps: (i) an update to *Z* given fixed Lagrange multipliers and (ii) an update to the Lagrange multipliers given a fixed *Z*. First, we find the unconstrained optimum ${\hat{Z}}_{:,j}^{*}$ using the algorithm described above. This value may not satisfy the non-negativity constraint. We compute the direction toward the optimum $s={\hat{Z}}_{:,j}^{*}-Z_{:,j}$. We find the largest value of *τ*_max_ such that $\Theta_{:,j}+\tau_{\text{max}}s$ satisfies the non-negativity constraint, and perform the update $Z_{:,j}^{*}\leftarrow Z_{:,j}+\tau_{\text{max}}s$. Second, we update the Lagrange multipliers. To do this, we maintain an active set *a* of the parameters with associated non-zero Lagrange multipliers. We update these Lagrange multipliers as follows.

(2) $$Z_{a,j}=0={\left(\sum_{g}\alpha_{g}\alpha_{g}^{T}\right)}_{a,a}^{-1}\left(\sum_{g}\alpha_{g,a}Y_{g,j}^{T}+\frac{1}{2}\Lambda_{j,a}^{T}\right)$$

(3) $$+{\left(\sum_{g}\alpha_{g}\alpha_{g}^{T}\right)}_{a,‒a}^{-1}\left(\sum^{g}\alpha_{g,‒a}Y_{g,j}^{T}\right)$$

(4) $$\Rightarrow \Lambda_{j,a}=-2\sum_{g}\alpha_{g,a}Y_{g,j}^{T}$$

(5) $$-2{\left(\sum_{g}\alpha_{g}\alpha_{g}^{T}\right)}_{a,a}{\left(\sum_{g}\alpha_{g}\alpha_{g}\right)}_{a,-a}^{-1}\left(\sum_{g}\alpha_{g,-a}Y_{g,j}^{T}\right).$$

Then, we start the next iteration with updated Lagrange multipliers.

We also developed a version of our model, *epigenome-ssm-sumone*, which incorporates a constraint that requires the chromatin state feature vector to sum to one (analogous to a probability vector). This model is the same as *epigenome-ssm-nonneg*, except that it includes an additional constraint, α≤1. We optimize this objective using the same active set method of Lagrange multipliers.

### 3.5 Alternative models

We compared *epigenome-ssm* with two well-known SAGA methods, ChromHMM and Segway. For ChromHMM, we used alignment files (.bam) from the Roadmap epigenomics portal (Roadmap-Epigenomics, 2020c) and used the *BinarizeBed* command provided in the ChromHMM package to binarize the data. We used ChromHMM *v1.22* (Ernst and Kellis, 2020) with the default parameters and the *printposterior* flag to save the posterior probabilities, which we used for the ChromHMM-con model (described below). As the name suggests, ChromHMM is based on HMM which generates a vector of *K* posterior probabilities for each genomic position and assigns the label with the highest probability to that position—a model which we call ChromHMM-dis in our evaluations. To adapt ChromHMM to produce continuous features, we also considered a variant of ChromHMM (which we term ChromHMM-con) that outputs the vector of continuous posterior probabilities to each genomic position. In practice, ChromHMM is extremely confident in its predictions, so ChromHMM-con’s features are mostly close to zero or one.

For Segway, we used the fold-change signal data from the Roadmap epigenomics portal (Roadmap-Epigenomics, 2020a). We used *genomedata v1.4.4* (Hoffman *et al.*, 2010) to create a genomedata archive for input to Segway. We used *Segway v3.0.2* with default parameters. We do not have Segway’s posterior probabilities to be used as continuous annotations in our evaluations because there was an issue with the posterior output in concatenated mode at the time that we performed our experiments.

### 3.6 Gene expression evaluation

We evaluated annotations according to their association with gene expression. In a high-quality annotation, highly expressed genes should be annotated in a distinct way from the rest of the genome. In other words, there should be a strong correlation between the annotation within the gene’s body and the gene’s expression level, as measured by RNA-seq.

**Fig. 2.**
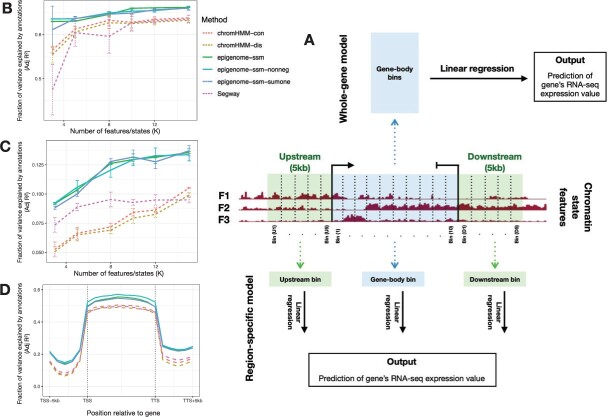
Evaluation of annotations generated by each method with respect to gene expression (as described in Section 3.6) and enhancer activity (as described in Section 3.7). (**A**) Schematic of gene expression evaluation. We divide each gene’s body (i.e. the region between TSS and TTS) into 10 bins of equal length. Also, we divide the regions 5 kb upstream of TSS and 5 kb downstream of TTS into five bins respectively. For the whole-gene model, we take the average value of features in each bin and concatenate them to form a feature vector for that gene. For instance, if the model has three features (as in this figure), the resulting vector will have 30 elements. We use the feature vector as predictor and the gene’s RNA-seq expression value as response for linear regression. The region-specific model is similar to the whole-gene model except that we use 20 linear regression models (one for each bin in the [TSS-5kb, TTS + 5kb] region) where each model takes as input the average feature values for its respective bin. (**B**) Evaluation of annotations relative to gene expression using the whole-gene model. (**C**) Similar to (B) but the evaluation is with respect to enhancer activity. (**D**) Evaluation of annotations relative to gene expression using the region-specific model

Following previous work (Libbrecht *et al.*, 2019; Zhang *et al.*, 2016; Zhang and Hardison, 2017), we evaluated an annotation according to the strength of correlation between the labels (or features, in the case of continuous annotation) within the body of a given gene and that gene’s expression. We downloaded RNA-seq data from the Roadmap Epigenomics data portal (Roadmap-Epigenomics, 2020b). The dataset contains information including location and expression level of 19 802 genes for 57 epigenomes. We included seven epigenomes in our evaluations (see Supplementary Table S1 for details) which gave us a total of 138 614 gene expression data points. We used a linear regression model to evaluate the degree to which annotations at a gene’s body are predictive of gene expression (Fig. 2A). We trained two types of prediction models: region-specific models and a whole-gene model.

The region-specific model evaluates the correlation between a gene’s expression and the chromatin state features at a given position relative to that gene. For each gene, we extracted annotations for the region between the following two positions: 5 kb upstream of transcription start site (TSS-5kb) and 5 kb downstream of transcription termination site (TTS + 5kb). We divided the [TSS-5kb, TSS], [TSS, TTS] and [TTS, TTS + 5kb] regions into 5, 10 and 5 evenly spaced bins respectively, for a total of 20 bins. For each bin, we trained a linear regression model. As the regression feature vector, we used the average feature vector in the respective bin. For discrete annotations, we used a one-hot encoding—that is, the feature vector has a 1 in the position corresponding to the label and 0’s elsewhere. As the regression response value, we used the RNA-seq RPKM (reads per kb per million mapped reads) values. We also transformed RPKM values with an arcsinh transformation. We used the fraction of variance explained (*R*^2^, also known as the coefficient of determination) to measure the predictive power of a regressor. To control for the complexity of the regressor, we used the standard adjustment $R^{2}=1-(1-r^{2})(n-1)/(n-p-1)$ (Dodge, 2008), where *n* and *p* are the number of examples and regressor parameters respectively.

For the whole-gene model, we applied another linear regression model on the entire gene body region [TSS, TTS]. This model is identical to the region-specific models, except that we take the average feature vectors only from bins within the gene body and use a single linear regression model to predict the gene’s RNA-seq expression value. The package *SigTools* (Masoumi *et al.*, 2022) was used to create some of the figures for this evaluation.

### 3.7 Enhancer evaluation

We additionally evaluated annotations according to their association with enhancer activity. In a high-quality annotation, highly active enhancers should be annotated in a distinct way from the rest of the genome. In other words, there should be a strong correlation between the annotation at the enhancer region and the enhancer’s activity level.

We used enhancer RNA (eRNA) as measured by Cap Analysis of Gene Expression (CAGE) as a proxy for enhancer activity, which we downloaded the from FANTOM5 website (FANTOM5, 2020). The dataset contains information such as location and activity level of 65 423 enhancers. Note that, because eRNAs are transcribed from the enhancer itself, the target gene of each enhancer is unknown. We included seven epigenomes in our evaluations (see Supplementary Table S1 for details) which gave us a total of 457 961 enhancer activity data points.

Following previous work (Zhang and Hardison, 2017), we evaluated an annotation according to the strength of correlation between the labels (or features, in the case of continuous annotation) within an enhancer region and the strength of activity of that enhancer. The model used for this analysis is similar to the whole-gene model for gene expression evaluation—we divide the enhancer region into 10 bins of equal size and use the average feature values within those bins as input to a linear regression model. As the regression response value, we took the arcsinh transformation of average TPM (tags per million mapped reads) values from the replicates that we used for each epigenome (See Supplementary Table S1 for data accession ids).

### 3.8 Prediction of genomic elements

We additionally evaluated chromatin state features according to how predictive they are of certain genomic elements. We evaluated according to two categories of elements: transcription start sites (TSSs) and enhancers, defined as described above. For each target element, we assigned a binary (0/1) response label to each position of the genome based on whether or not that position belongs to that particular element. We used each chromatin state feature in turn as a prediction score, analogous to a prediction probability output from a classifier. We defined a receiver operating characteristic (ROC) curve for each element–feature pair by varying the threshold on the chromatin state feature used as positive predictions.

## 4 Results

### 4.1 Chromatin state features at genes are predictive of gene expression

To evaluate *epigenome-ssm* and its variants, we used the resulting annotations to predict RNA-seq gene expression data, following previous work (Libbrecht *et al.*, 2019; Zhang *et al.*, 2016) (Section 3.6). Briefly, we used a linear regression model to evaluate the degree to which annotations at a gene region are predictive of gene expression. We computed the average feature vector over the entire gene region [TSS, TTS]. As the regression response value, we used the RNA-seq RPKM (reads per kb per million mapped reads) values. We used the fraction of variance explained (adjusted *R*^2^, also known as the coefficient of determination) to measure the predictive power of a regressor.

We found that all methods are predictive of gene expression, but SSM-based models (epigenome-ssm-*) clearly outperform alternatives by this measure in all cases of *K* (Fig. 2B). An epigenome-ssm-nonneg annotation with *K *=* *5 explains more variance (Adj *R*^2^ = 0.64) than the discrete ChromHMM and Segway (Adj *R*^2^ = 0.60 and 0.60 respectively). Part of this improvement is a result of the greater richness of a continuous model—if we produce a continuous annotation from the ChromHMM by using the probability of each state (ChromHMM-con), the performance improves (Adj *R*^2^ = 0.60 and 0.61 for ChromHMM-dis and ChromHMM-con respectively). However, performance of ChromHMM-con is still worse than the *epigenome-ssm* annotation, indicating the importance of using a model that is intrinsically continuous.

Moreover, the superior performance of the epigenome-ssm-* is maintained even when ChromHMM and Segway use more labels than the number of SSM features. Even for the largest number of labels we tried (*K *=* *15), the performance of ChromHMM and Segway models were no higher than Adj *R*^2^ = 0.63, which is lower than that of *epigenome-ssm-nonneg* with just 5 features ($\mathrm{Adj}R^{2}$ = 0.64). This comparison offsets the potential disadvantage that continuous features are more complex than discrete labels. If one is interested in obtaining a very simple annotation, according to this analysis, it is preferable to use a continuous model with a small number of features rather than a discrete model with many labels. Adding the non-negativity or sum-to-one constraints to *epigenome-ssm* does not significantly change prediction performance, while improving interpretability.

To put this improvement in *R*^2^ in perspective, we found that this improvement is comparable to the gain in performance from adding three additional input datasets. First, take for example the gene expression evaluation using the whole-gene model (Fig. 2B). We repeated the whole-gene expression evaluation (with *epigenome-ssm-nonneg* and *K *=* *5) 12 times, each time removing one of the 12 input tracks from training. On average, removing a single track decreases the *R*^2^ by 0.0135 (Supplementary Fig. S8). This difference is approximately one-third of the difference in *R*^2^ between *epigenome-ssm-nonneg* ($R^{2}=0.64$) and Segway ($R^{2}=0.60$).

We additionally tested several of alternative approaches for defining chromatin state features (Supplementary Fig. S6): principle component analysis (PCA), non-negative matrix factorization (NMF) and four variants of a hidden Markov model (HMM) that can be thought of as generalizations of Segway and ChromHMM. We found that the increased performance of epigenome-ssm-* is maintained relative to these alternative approaches.

To evaluate which positions relative to each gene are most predictive of expression, we divided the [TSS-5kb, TTS + 5kb] region into multiple equal-sized bins and trained a region specific prediction model for each bin (Fig. 2D). As we expected, positions within the gene body are most predictive of expression, whereas annotations at positions over 3 kb from the TSS have less predictive power. Also, *epigenome-ssm* models are consistently better in predicting gene expression throughout the [TSS-5kb, TTS + 5kb] region compared to both ChromHMM and Segway.

### 4.2 Chromatin state features at enhancer elements are predictive of enhancer activity

We further evaluated these annotation methods by measuring how predictive each annotation is of experimentally validated enhancer elements, again following previous work (Zhang *et al.*, 2016). As illustrated by Figure 2C, the three variants of *epigenome-ssm* perform significantly better than both ChromHMM and Segway in this task. Similar to the gene expression evaluation, *epigenome-ssm* maintained its superior performance even when ChromHMM and Segway use more labels than the number of SSM features. Even for the largest number of labels we tried (*K *=* *15), the performance of ChromHMM and Segway models were no higher than *R*^2^ = 0.11, which is lower than the performance of the *epigenome-ssm* model with 8 features (*R*^2^ = 0.12).

Similar to the gene expression evaluation, adding the non-negativity and sum-to-one constraints to *epigenome-ssm* did not significantly impact the performance. Overall, the performance of the constrained models is superior to ChromHMM and Segway.

### 4.3 Chromatin state features are predictive of genomic elements

We further evaluated chromatin state features according to how accurately they identify genomic elements such as transcription start sites (TSSs) and enhancer regions. To do so, we used a given feature as a score (analogous to a prediction probability output from a classifier) and calculated the area under the resulting receiver operating characteristic (ROC) curve (Section Prediction of genomic elements). For each genomic feature type (TSSs and enhancers), we selected the feature from each model with the highest area under the ROC curve (auROC).

**Fig. 3.**
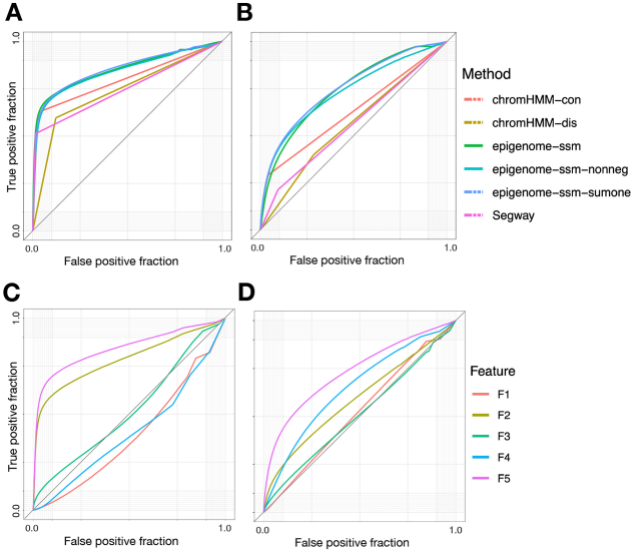
(**A, B**) Receiver operating characteristic (ROC) curves indicating accuracy with which a given model’s best feature predicts (A) transcription start sites (TSSs) and (B) enhancer regions. epigenome-ssm features have higher area under the curve (AUC) for both TSSs and enhancers compared to Segway and ChromHMM features. Also, ChromHMM-con’s curves bend sharply because this model is extremely confident in the labels it generates which results in having the posterior probabilities being mostly close to zero or one. (**C, D**) ROC curve indicating accuracy with which each feature predicts (C) transcription start sites (TSSs) and (D) enhancer regions. A curve that falls below the *y *=* x* line indicates that higher values of the feature are depleted for that particular element

We found that epigenome-ssm-* features achieve much superior auROC than alternatives (Fig. 3A and B). For example, *epigenome-ssm-nonneg* F5 achieves an auROC of 0.84 for identifying TSSs, compared to an auROC of 0.80 for ChromHMM-con’s best label, L1 (Fig. 3A). This improved performance results primarily from the fact that a continuous feature can trace out the tradeoff between false positives and false negatives, leading to a smooth ROC curve. In contrast, a discrete prediction corresponds to a single point on the ROC curve. Note that even though ChromHMM-con outputs continuous posterior probability values, these probabilities are almost all either zero or one, resulting in the same shape of ROC curve.

### 4.4 Chromatin state features recapitulate known genome biology

**Fig. 4.**
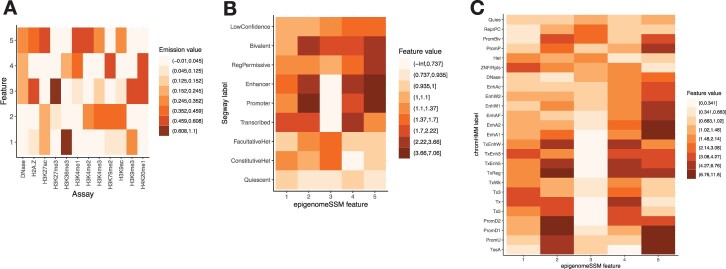
Visualization of model parameters and chromatin state features generated by an *epigenome-ssm-nonneg* model with *K *=* *5. (**A**) Emission matrix of the model: it shows the relationship of features to the input assays. Color corresponds to the mean signal value of a given assay at positions annotated with a given feature. (**B, C**) Average value of each SSM feature at each (B) Segway and (C) ChromHMM label

**Fig. 5.**
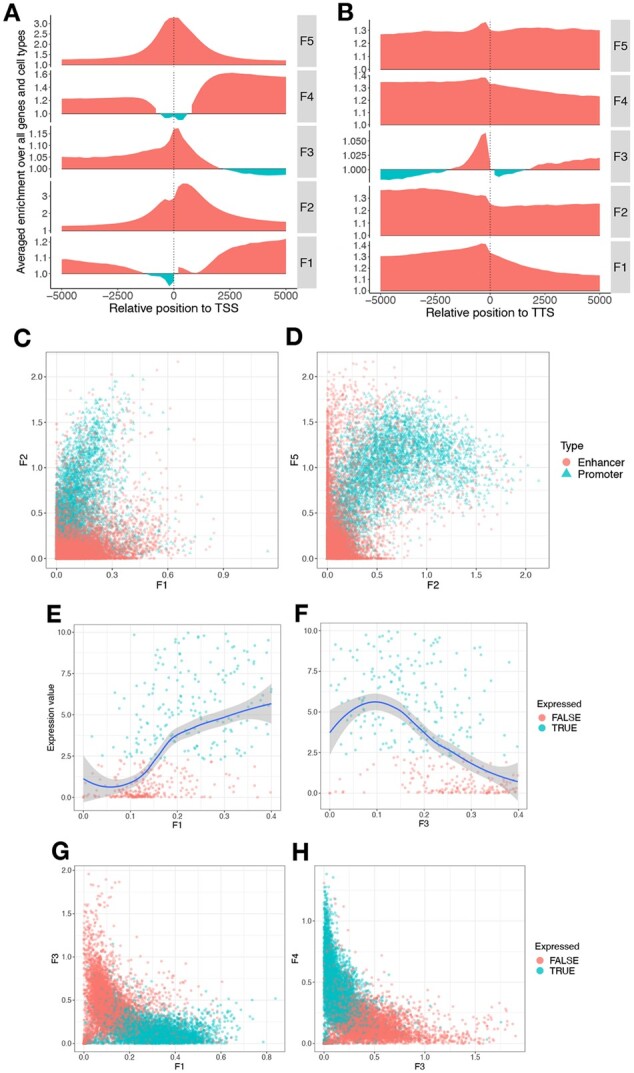
Visualization of chromatin state features generated by an *epigenome-ssm-nonneg* model with *K *=* *5. (**A, B**) Averaged enrichment of features with relative position to (A) TSS (B) TTS. Feature values are normalized by dividing them by the genome-wide mean for each feature. (**C, D**) Distribution of features at enhancer and promoter regions. Each point corresponds to either an enhancer or promoter depending on its color. (**E, F**) Relationship of features within the gene body with gene expression. Each point corresponds to a gene and the color of each point indicates whether or not the corresponding gene is expressed. As a threshold for whether or not to consider a gene as expressed, we took the median expression level of all the genes for each epigenome and set the average of the medians as the threshold. (**G, H**) Features within gene body for pairs of features. Each point corresponds to a gene and the color of each point indicates whether or not the corresponding gene is expressed. The complete set of plots for each evaluation can be found in the Supplementary Material (Supplementary Figs S12–S14)

While the results above show quantitatively that chromatin state features are predictive of many genomic phenomena, we additionally found that these features qualitatively recapitulate known genome biology (Figs 4 and 5). We focus here on an annotation generated by *epigenome-ssm-nonneg* using five features (*K *=* *5). These five features summarize the chromatin state represented by 12 epigenomic tracks; characterizing such state using a discrete SAGA annotation would require 10–20 labels.

We chose the non-negative model because it is easier to interpret than a model with both negative and positive features. We chose non-negative over sum-to-one because we found that the sum-to-one model usually outputs one ‘quiescent’ feature with a zero-valued emission vector, which essentially reduces the sum-to-one model to a non-negative model with one fewer feature.

The *epigenome-ssm-nonneg* model identifies two types of regulatory activity specific to promoters and enhancers respectively. Feature 2 is a mark of promoter activity, characterized by the histone modifications H3K4me3 and H3K9ac (Fig. 4A). Likewise, F2 has a high value in the regions that are labeled as promoter by Segway and ChromHMM respectively (Fig. 4B and C). F2 is highly predictive of transcription start sites (Fig. 3C). Note that F2 has a low emission for general regulatory marks common at promoters, such as DNase-seq and H3K4me1, because those are also marked by F5.

Feature 5 is a mark of general regulatory activity, characterized by the regulation-associated histone modifications H3K4me1 and H3K27ac (Fig. 4A). F5 is present at both promoters and enhancers; promoters are characterized by high values of both F2 and F5 (Figs 3C, 4B, C, 5A and C), whereas enhancers are marked with just F5 (Figs 3C, 4B, C and 5D). Characterizing promoters and enhancers this way accurately characterizes elements into a continuum of ‘promoter-ness’ (F5), rather than as two disjoint categories.

Features 1 and 4 mark transcribed genes. Feature 4 is characterized by histone modifications H3K36me3, H4K20me1 and H3K79me2 (Fig. 4A). Hence, this feature is found downstream of TSS, within the gene body (Fig. 5A), and has high values at regions labeled as transcribed (‘Transcribed’ or ‘TxXX’) by Segway and ChromHMM (Fig. 4B and C). Feature 1 is characterized by the histone modification H3K36me3 (Fig. 4A) and is specific to the later parts of the gene body (Figs 4C and 5B). Both F1 and F4 over the gene body are highly correlated with RNA-seq expression (Fig. 5E and Supplementary Fig. S13D).

Feature 3 is a mark of repression, characterized by the repressive histone modifications H3K27me3 and H3K9me3 (Fig. 4A). F3 at the gene body has a strong negative correlation with gene expression (Fig. 5F). Note that F3 encompasses both facultative (polycomb, H3K27me3) and constitutive (H3K9me3) heterochromatin, which usually occur in different genomic regions. These two types separate into separate features when using a *epigenome-ssm-nonneg* model with eight features (Supplementary Fig. S10).

Notably, *epigenome-ssm-nonneg* does not include features corresponding to ‘Weak Enhancer’ or ‘Weak Transcription’ commonly present in other SAGA methods (Fig. 4B and C). Instead, the strength of an element is represented simply by the feature value itself. Representing element strength this way reveals trends that are hidden by a discrete annotation. For example, the strength of transcription-related marks (F1,4) is correlated with expression, but with diminishing returns (Figs 5E and Supplementary Fig. S13D). Conversely, while strong repressive marks (F3) are correlated with low expression, moderate-strength repressive marks have no impact on expression (Fig. 5F).

Similarly, *epigenome-ssm-nonneg* does not include features that correspond to the ‘Quiescent’ label commonly present in SAGA methods. Such regions are represented simply by an absence of the other features (Fig. 4B and C).

The *epigenome-ssm-nonneg* model can easily represent mixtures of activity types. For example, other SAGA methods commonly report a ‘Bivalent Promoter’ label characterized by both activating and repressive marks. Such regions are labeled by *epigenome-ssm-nonneg* simply with a mixture of active (F2/5) and repressive (F3) features (Fig. 4B and C).

### 4.5 Chromatin state features enable visualization

Chromatin state features easily lend themselves to expressive visualizations that can be very useful in several downstream analyses. For instance, in Figure 5D, enhancers and promoters are clearly separated in the space of feature 2 and feature 5. This further confirms the interpretation in the previous section that feature 2 and 5 are marks of promoter and general regulatory activity respectively. Likewise, Figure 5E illustrates the strong positive correlation of feature 1 with gene expression, while Figure 5F shows that feature 3 is negatively correlated with gene expression. Moreover, the scatter plots in Figure 5G and H show how genes are clustered in the space of features 1, 3 and 4 based on whether or not they are expressed.

Summarizing genomic activity into a small set of chromatin state features is particularly important for visualization because plots have limited dimensions (e.g. x/y axes) onto which to map features. For example, one can visualize all the variation in five features using just ten pairwise scatterplots (Supplementary Figs S12 and S14). In contrast, doing so with 12 tracks would require 66 such plots.

## 5 Discussion

In this work, we introduced continuous chromatin state features for genome annotation. These chromatin state features are analogous to existing discrete chromatin state labels, but continuous features have several benefits: they can represent varying strength among elements, and they can easily represent combinatorial patterns of activity. Due to these benefits, we showed that chromatin state features outperform existing discrete annotations at predicting gene expression and enhancer activity.

Continuous chromatin state features present an alternative representation of genomic activity to existing SAGA labels. We expect that both types of annotations will be used in practice. Discrete labels are most effective when a fixed set of elements is needed. However, we expect that continuous features will be used in applications where the limitations of discrete labels make such labels ineffective.

In particular, chromatin state features are useful for producing expressive visualizations. We showed that visualizing chromatin state features from *epigenome-ssm* correctly depicts the continua of expressed to not-expressed genes and promoters to enhancers. Such a continuum is impossible to express in a discrete framework, which must use hard thresholds. Moreover, although each continuous feature is more complicated to interpret than a discrete label, we showed that a small number of continuous features outperform even a large number of discrete labels in all of our evaluations. Therefore, a small number of chromatin state features can replace a much larger number of discrete labels, decreasing the overall complexity of the annotation.

Because continuous annotations maintain much more of the information in the input data than discrete annotations do, they are more useful for complex downstream applications. For example, a variant effect predictor might take chromatin state features as input in order to predict the functional impact of a given mutation. This is preferable to using raw tracks for two reasons. First, a small number of chromatin state features concisely summarize a large number of input tracks and therefore a predictive model based on these features will be less prone to overfitting. Second, chromatin state features can be used for variant effect interpretation; that is, a model could report that its prediction of high variant effect is due to the fact that a specific feature is present at that position. Such interpretation is more difficult with raw tracks because most types of activity are associated with a combination of many marks. In the future, we plan to apply this approach at a large scale to create reference chromatin state feature annotations for all tissues with sufficient available data.

## Funding

This work was supported by NSERC Discovery Grants numbers 611362 and 06150, a MSFHR Scholar Award, Compute Canada RRG kdd-445-01 and Genome Canada/Genome BC 283BAC.

*Conflict of Interest*: none declared.

## Supplementary Material

btac283_Supplementary_Data
