# Correction to: Continuous chromatin state feature annotation of the human epigenome

**DOI:** 10.1093/bioinformatics/btad163

**Published:** 2023-04-24

This is a correction to: Habib Daneshpajouh, Bowen Chen, Neda Shokraneh, Shohre Masoumi, Kay C Wiese, Maxwell W Libbrecht, Continuous chromatin state feature annotation of the human epigenome, Bioinformatics, Volume 38, Issue 11, 1 June 2022, Pages 3029–3036, https://doi.org/10.1093/bioinformatics/btac283

An update has been made to the Funding section of the online version of this manuscript.

In the originally published version, the Funding section was given as follows:

This work was supported by NSERC Disccovery Grants numbers 611362 and 06150, a MSFHR Scholar Award and Compute Canada RRG kdd-445-01.

This has been corrected online to:

This work was supported by NSERC Discovery Grants numbers 611362 and 06150, a MSFHR Scholar Award, Compute Canada RRG kdd-445-01 and Genome Canada/Genome BC 283BAC.

