# Supplementary material for: Continuous chromatin state feature annotation of the human epigenome
Source: Bioinformatics. 2022 Apr 22;38(11):3029–36. doi: 10.1093/bioinformatics/btac283 (PMC9154241; doi:10.1093/bioinformatics/btac283)
Supplement: btac283_Supplementary_Data [file btac283_supplementary_data.zip › 2022-02-18_daneshpajouh_continuous_supplement.pdf]

Supplementary material

Hidden Markov model (HMM) and other alternative models

The most common model used in previously-proposed genome annotation methods is the hidden Markov model (HMM). The HMM assumes that at position g there is a latent state $x_g \in \{1 \ldots K\}$ that represents the chromatin state label of that position. It assumes that the observed data is generated as a function of the latent state x_g (see next paragraph). It further assumes that the state at position g depends just on the state at position $g-1$, and that state l transitions to state l' with probability $\phi_{\ell,\ell'}$

$$P(x_g = \ell' | x_{g-1} = \ell) = \phi_{\ell,\ell'} \qquad \sum_{\ell'=1}^{K} \phi_{\ell,\ell'} = 1 \tag{6}$$

We considered two versions of the HMM model, which take as input continuous or discrete data respectively. As described in the Related work section, some existing methods take continuous input [3, 16] while others take discrete input [13, 9]. In the continuous input case, as with the state space model, the continuous-input HMM takes as input a vector of m observed genomic data sets for each position, $y_g \in \mathbb{R}^m$, for $g \in 1 \ldots T$. It assumes that there is a mean vector associated with each state μ_k, and that the observed data vector equals μ_k plus Gaussian noise

$$y_g = \mu_{x_g} + \epsilon_g \qquad \epsilon_g \sim N(0, \Sigma^2_{x_g}). \tag{7}$$

In the discrete input case, input data is thresholded into binary values such that the input data at position g is represented by a binary vector $\bar{y}_g \in \{0,1\}^m$. The discrete-input HMM assumes that the observed data is generated as a multivariate Bernoulli distribution. That is, for track i at position g,

$$P(\bar{y}_{i,g} = 1) = \theta_{x_k,i}. \tag{8}$$

To learn the HMM model, we use the EM algorithm to maximize the log likelihood of the model as a function of the model's parameters: $\mu_{1:k}$, $\Sigma_{1:k}$ and $\phi_{1:k,1:k}$ for the continuous-input model, and $\theta_{1:m,1:k}$ and $\phi_{1:k,1:k}$ for the discrete-input model.

We consider two possible ways of representing the output of the HMM model. Existing methods generally output a discrete value for the inferred state x_g as the chromatin state label at position g. However, an HMM can be re-purposed to output continuous chromatin state features by defining the chromatin state feature ℓ at position g as $\alpha_{\ell,g} = P(x_g = \ell)$.

In summary, we have two choices for input (continuous input with a Gaussian distribution or discrete input with a Bernoulli distribution) and two choices for output (output continuous chromatin state features or discrete chromatin state labels), giving us four HMM variants: HMMgaus-con, HMMgaus-dis, HMMber-con and HMMber-dis. We used the Python package pomegranate [31] for all training and inference of HMM models.

We additionally employed two other alternative models. We investigated principle component analysis (PCA) [26] and non-negative matrix factorization (NMF) [34]. Both methods take as input a vector $y_g \in \mathbb{R}^m$ and output a vector of features $\alpha_g \in \mathbb{R}^K$ to optimize an objective function. They differ from the SSM and HMM methods in that PCA and NMF treat each position independently, without considering the genome coordinate. NMF differs from PCA in that it outputs non-negative values $\alpha_{i,t} \geq 0$. We used implementations of PCA and NMF from scikit-learn [27].

Fig. 6 shows the evaluation results of these alternative methods.

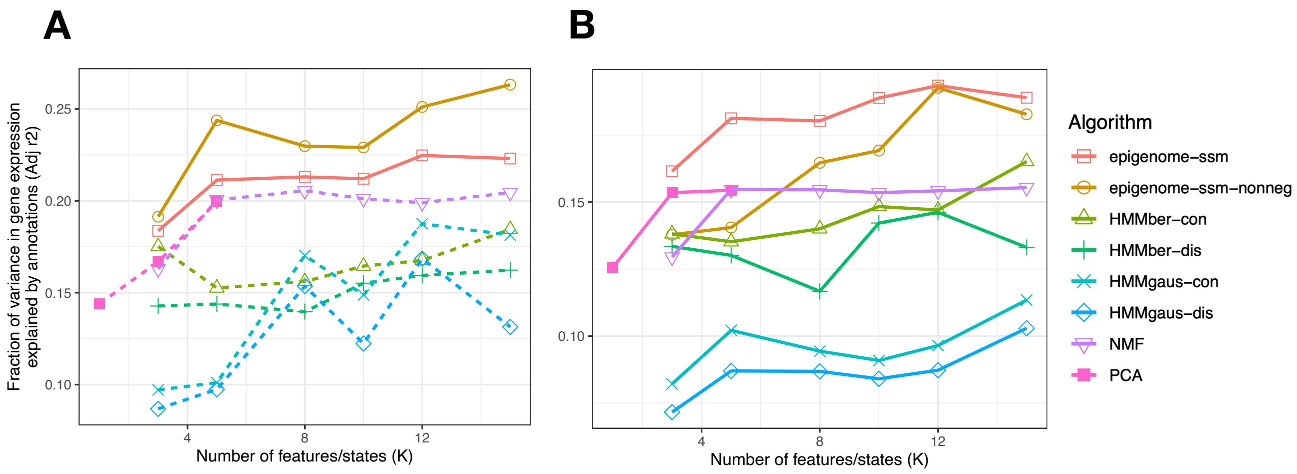

Fig. 6: (A) Evaluation of annotations relative to gene expression using the whole-gene model. (B) Similar to (A) but the evaluation is with respect to enhancer activity.

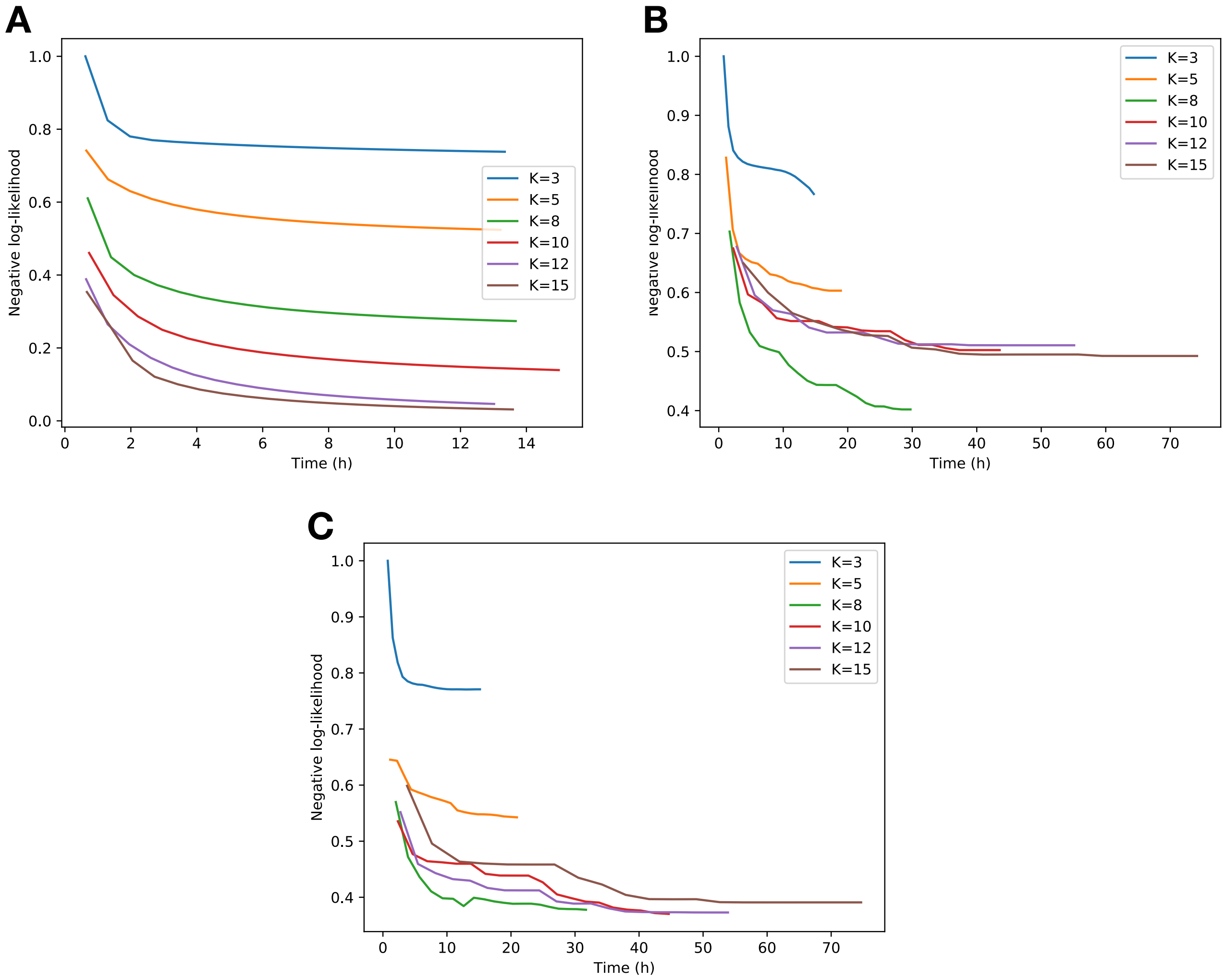

Fig. 7: The time taken for optimization of (A) epigenome-ssm, (B) epigenome-ssm-nonneg, and (C) epigenome-ssm-sumone models.

Table 1. Epigenomes (cell types) whose data are used as input to our models. The epigenome IDs are from Roadmap Epigenomics, while replicate IDs are from FANTOM5 enhancer data that we used for enhancer evaluation. Assay signal data form all the epigenomes were used as input to our models for training and annotations—however, E017 is excluded from evaluations because its RNA-seq and enhancer data are not available in the Roadmap Epigenomics and FANTOM5 databases.

Epigenome name	Roadmap epigenome ID	FANTOM5 replicate ID
H1 Cells	E003	CNhs14067, CNhs14068, CNhs13964
IMR90 fetal lung fibroblasts Cell Line	E017	* Excluded from evaluations
A549 EtOH 0.02pct Lung Carcinoma Cell Line	E114	CNhs11275
GM12878 Lymphoblastoid Cells	E116	CNhs12331, CNhs12332, CNhs12333
HUVEC Umbilical Vein Endothelial Primary Cells	E122	CNhs10872, CNhs11967, CNhs12010
K562 Leukemia Cells	E123	CNhs11250, CNhs12334, CNhs12335, CNhs12336
NHEK-Epidermal Keratinocyte Primary Cells	E127	CNhs11064, CNhs11381, CNhs12031
NHLF Lung Fibroblast Primary Cells	E128	CNhs12500, CNhs11380, CNhs12029

Table 2. Assays (epigenetic marks) whose data are used as input to our models.

Assay ID	Association
DNase	DNA accessibility
H2A.Z	Transcription activation and repression
H3K27ac	Activation; Enhancer; Promoter
H3K27me3	Repression; Promoter
H3K36me3	Activation; Transcribed region
H3K4me1	Poised enhancer
H3K4me2	Transcription
H3K4me3	Active promoter; Transcription
H3K79me2	Activation; Transcription; Gene body
H3K9ac	Activation; Enhancer; Promoter
H3K9me3	Repression; Heterochromatin
H4K20me1	Transcription

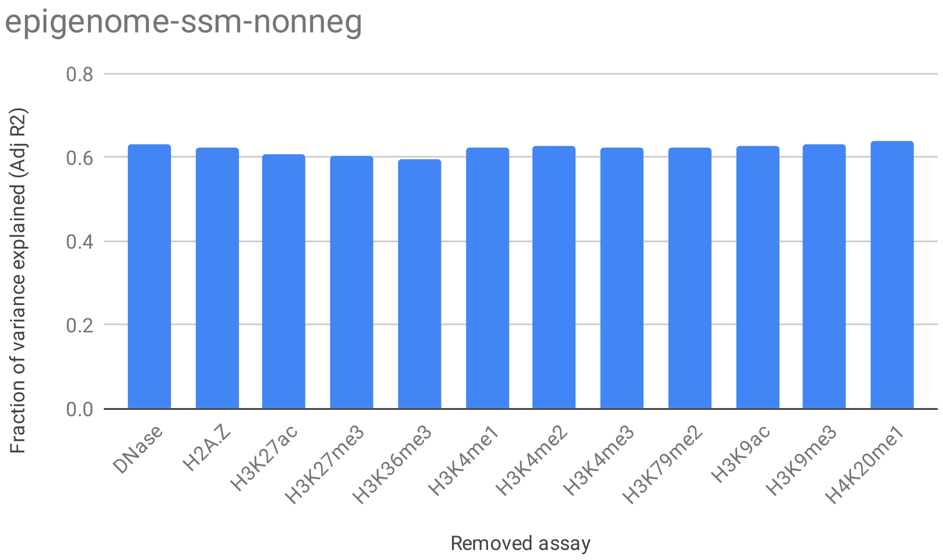

Fig. 8: Gene expression evaluation using the whole-gene model with *epigenome-ssm-nonneg* and $K = 5$. Each time, one of the input tracks shown on the horizontal axis is removed from training to see its impact on gene expression prediction.

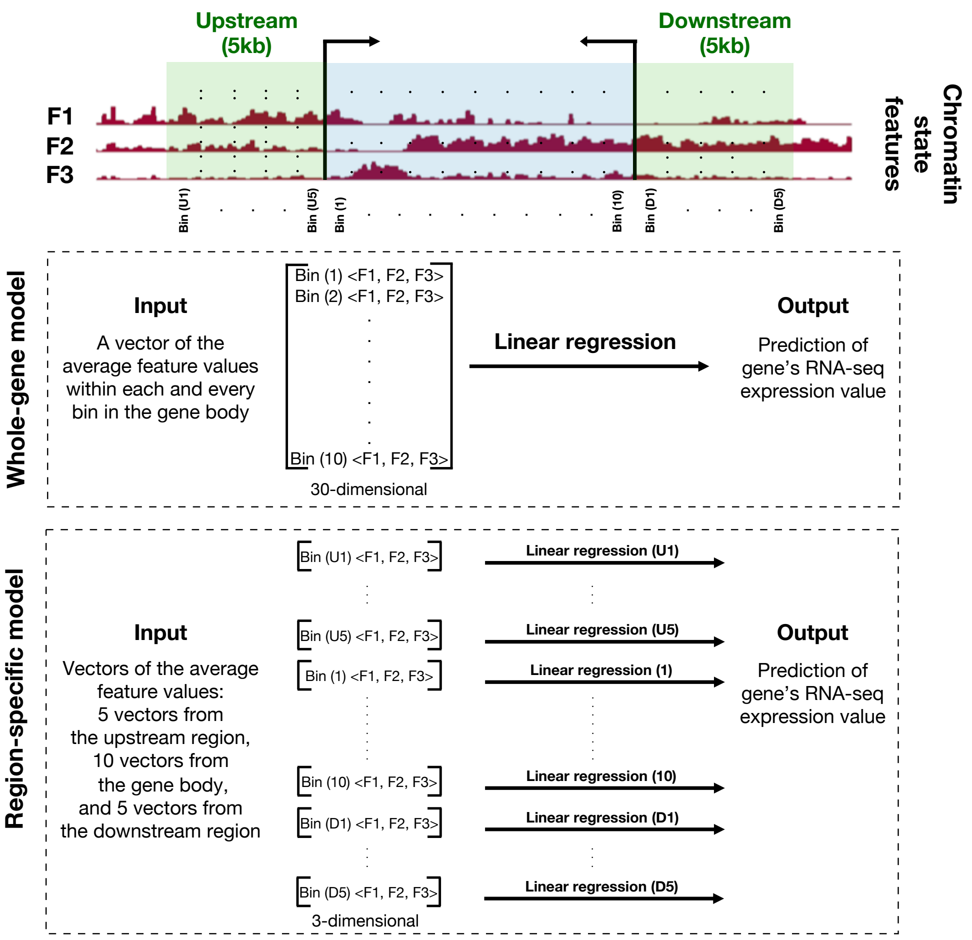

Fig. 9: Evaluation of annotations generated by each method with respect to gene expression (as described in Gene expression evaluation section) and enhancer activity (as described in Enhancer evaluation section). We evaluate the annotations according to the strength of correlation between the labels (or features, in the case of continuous annotation) within the body of a given gene and that gene’s expression. We divide each gene’s body (i.e. the region between TSS and TTS) into 10 bins of equal length. For the whole-gene model, we take the average value of features in each bin and concatenate them to form a feature vector for that gene. For instance, if the model has three features (as in this figure), the resulting vector will have 30 elements. We use the feature vector as predictor and the gene’s RNA-seq expression value as response for linear regression. The region-specific model is similar to the whole-gene model except that we use 10 linear regression models (one for each bin) where each model takes as input the average feature values for its respective bin.

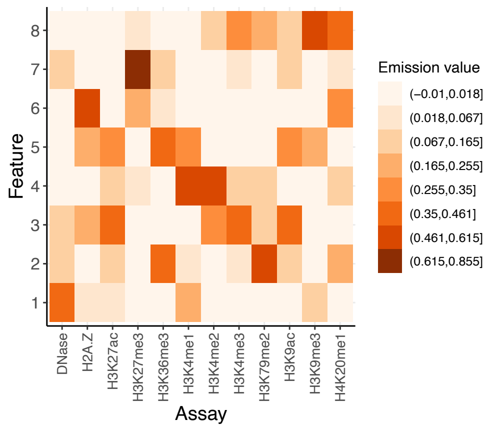

Fig. 10: Emission matrix of an *epigenome-ssm-nonneg* model with eight features: it shows the relationship of features to the input assays. Color corresponds to the mean signal value of a given assay at positions annotated with a given feature.

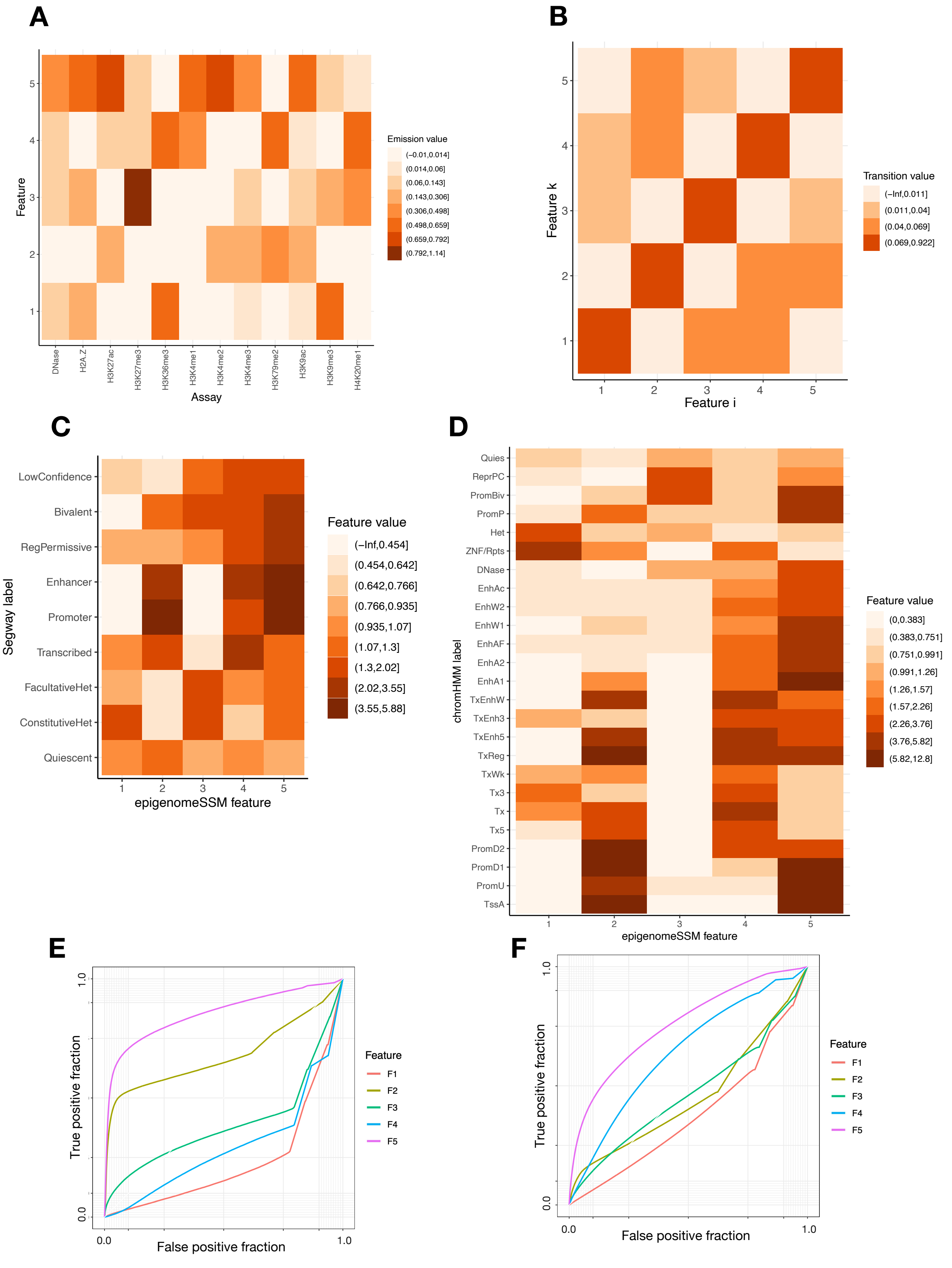

Fig. 11: Visualization of model parameters and chromatin state features generated by an *epigenome-ssm-sumone* model with $K = 5$ (similar to Fig. 4). (A) Emission matrix of the model: it shows the relationship of features to the input assays. Color corresponds to the mean signal value of a given assay at positions annotated with a given feature. (B) Transition matrix of the model: it shows the relationship between features in neighboring positions. Color in cell i, k represents the correlation of feature i at one position with feature k in the following position. (C-D) Average value of each SSM feature at each (c) Segway and (d) ChromHMM label. (E) ROC curves generated using features at TSS. (F) ROC curves generated using features within enhancer regions.

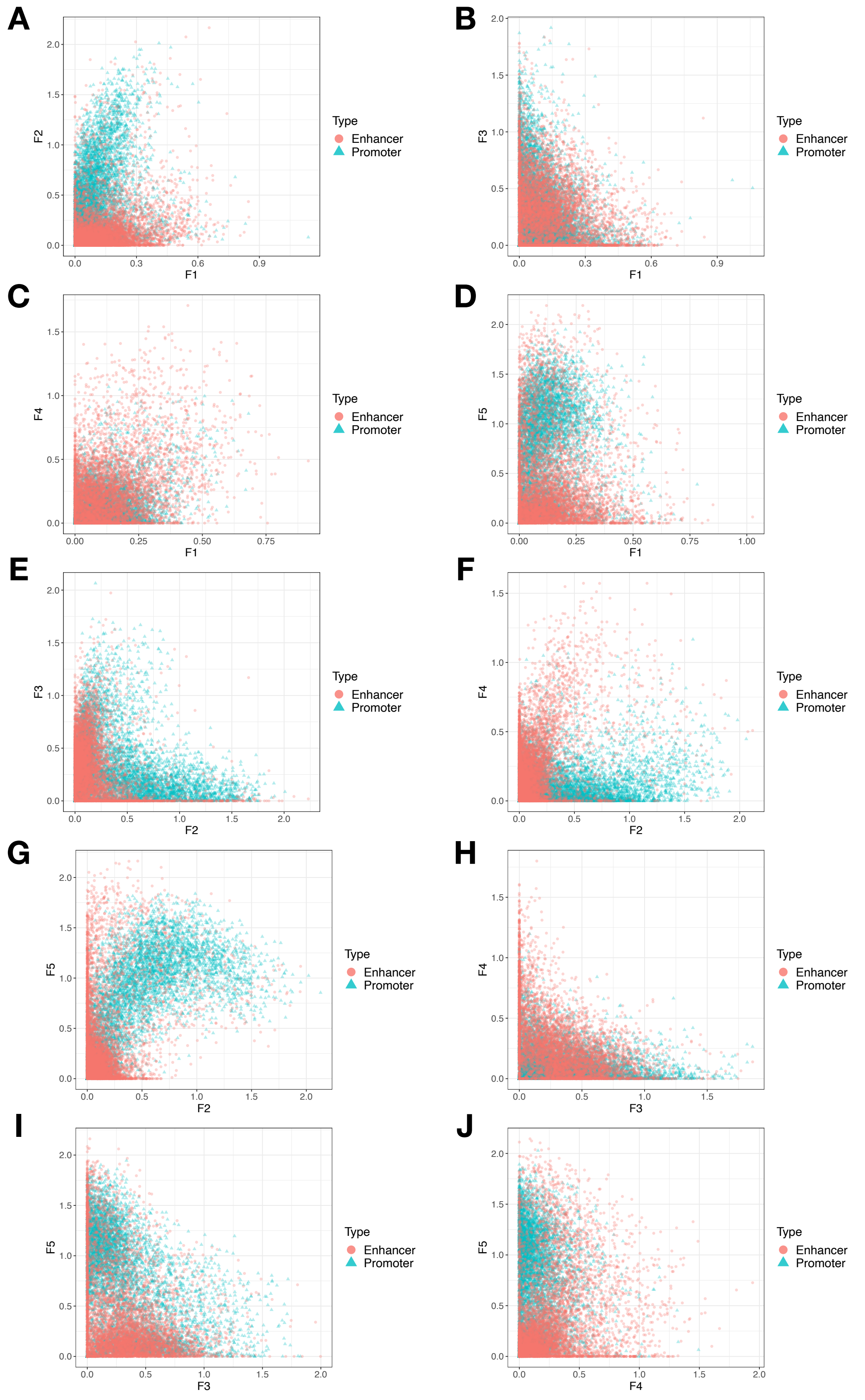

Fig. 12: Distribution of chromatin state features within enhancer and promoter regions (similar to Fig. 5C-D). The color and shape of each point indicate whether the corresponding element is an enhancer or a promoter. These plots show how enhancers and promoters can be distinguished using chromatin state features. The features are generated by an *epigenome-ssm-nonneg* model with $K = 5$.

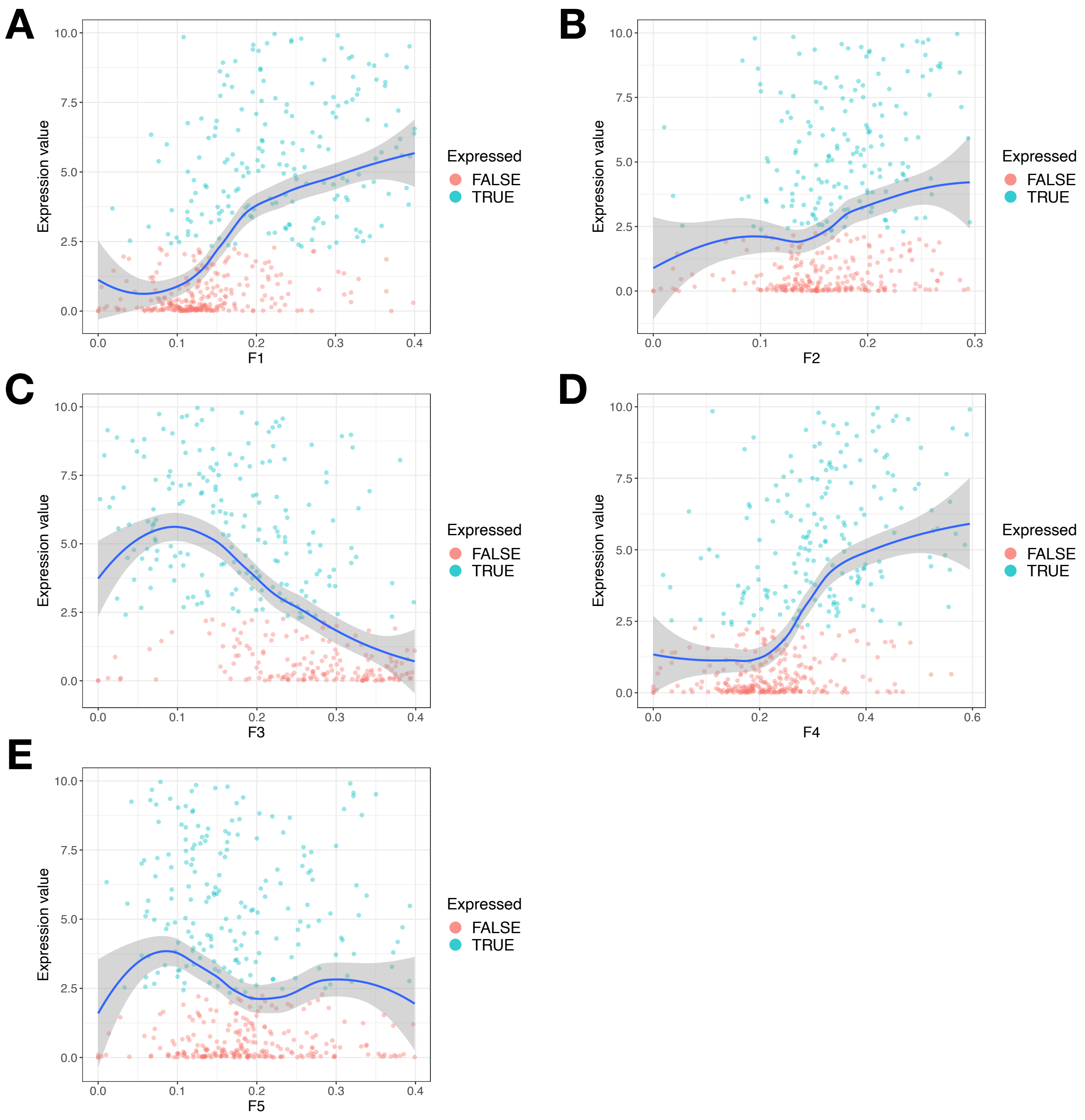

Fig. 13: Relationship of chromatin state features within the gene body with gene expression (similar to Fig. 5E-F). Each point corresponds to a gene and the color of each point indicates whether or not the corresponding gene is expressed. F1 and F4 show a strong positive correlation with gene expression while F3 has a negative correlation with gene expression. The features are generated by an epigenome-ssm-nonneg model with $K = 5$.

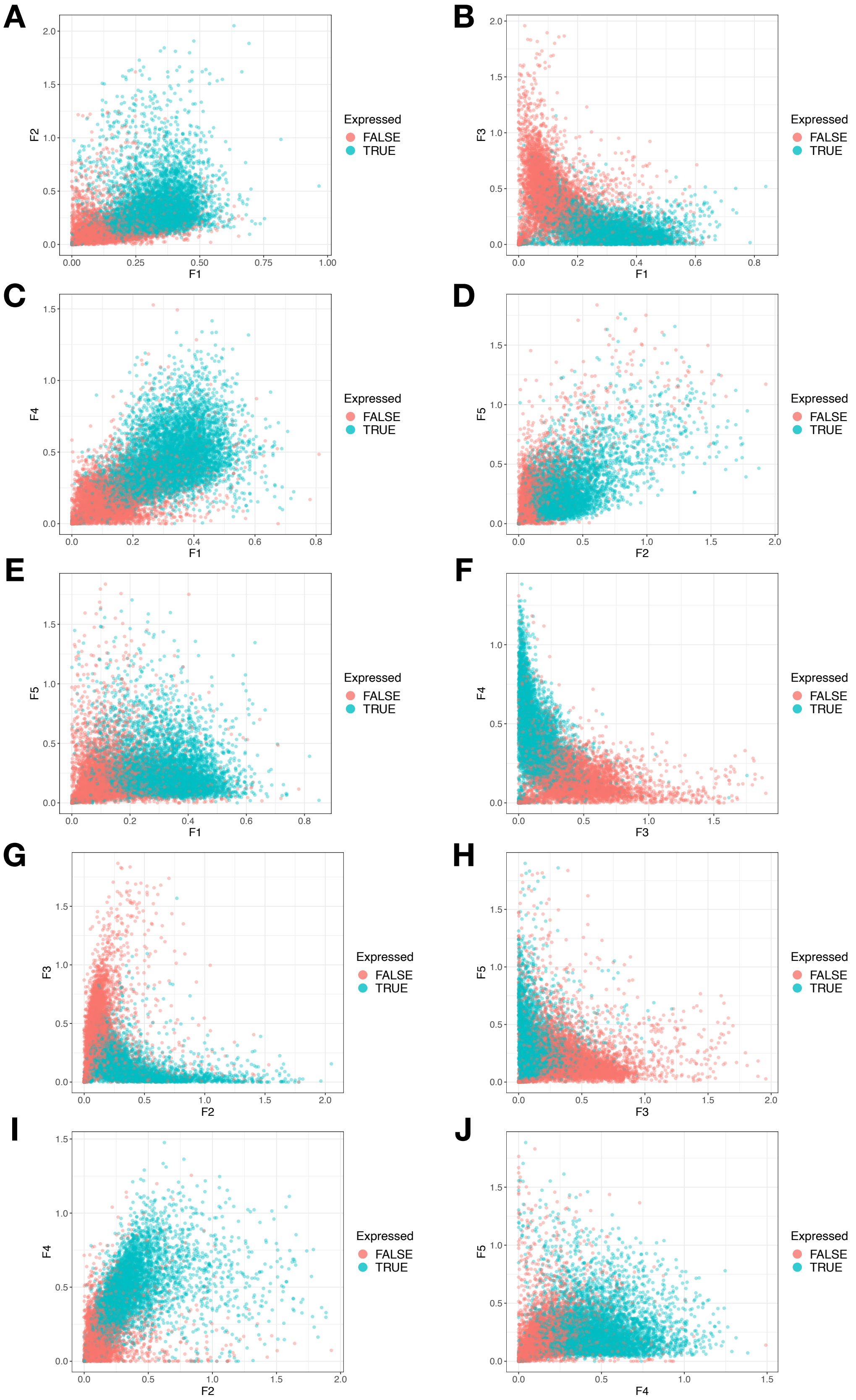

Fig. 14: Distribution of chromatin state features within gene body (similar to Fig. 5G-H). Each point corresponds to a gene and the color of each point indicates whether or not the corresponding gene is expressed. These plots show how expressed and not-expressed genes can be distinguished using chromatin state features. The features are generated by an epigenome-ssm-nonneg model with $K = 5$.

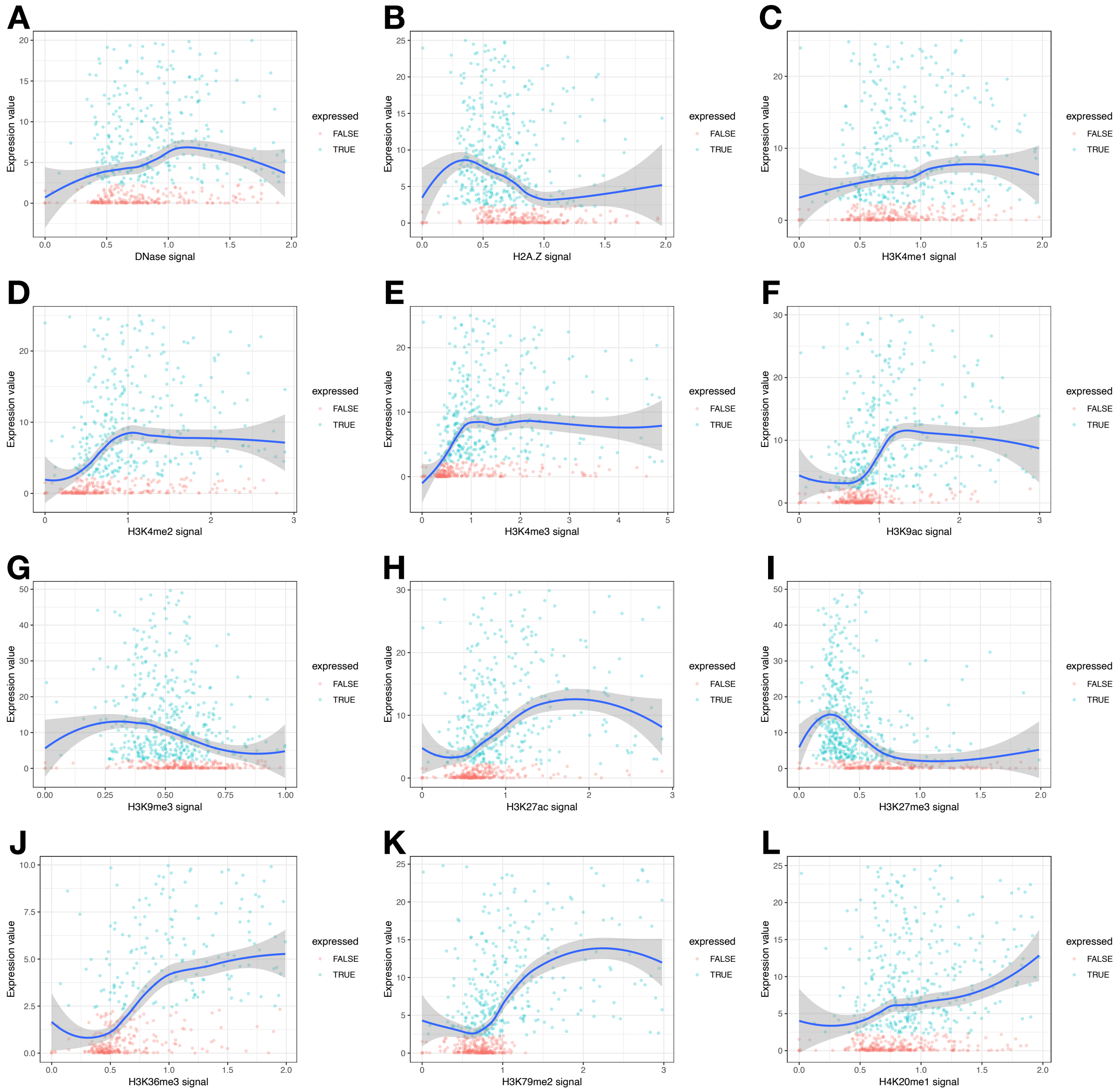

Fig. 15: The relationship of input assay signals used in our experiments with gene expression. Each point corresponds to a gene and the color of each point indicates whether or not the corresponding gene is expressed.
